# Grouping Digital Health Apps Based on Their Quality and User Ratings Using K-Medoids Clustering: Cross-Sectional Study

**DOI:** 10.2196/57279

**Published:** 2025-07-23

**Authors:** Maciej Marek Zych, Raymond Bond, Maurice Mulvenna, Lu Bai, Jorge Martinez-Carracedo, Simon Leigh

**Affiliations:** 1 School of Computing, Ulster University, 2-24 York Street, Belfast, BT15 1AP, United Kingdom, 44 7526852505; 2 School of Electronics, Electrical Engineering and Computer Science, Queen's University, Belfast, United Kingdom; 3 Organisation for the Review of Care and Health Apps (ORCHA), Daresbury, United Kingdom

**Keywords:** digital health, ORCHA assessment, quality assessment, cluster analysis, digital health app, app, user, clinical assurance, user experience, data privacy, mHealth, Organisation for the Review of Care and Health Apps, mobile health

## Abstract

**Background:**

Digital health apps allow for proactive rather than reactive health care and have the potential to take the pressure off health care providers. With over 350,000 digital health apps available on the app stores today, those apps need to be of sufficient quality to be safe to use. Discovering the typology of digital health apps regarding professional and clinical assurance (PCA), user experience (UX), data privacy (DP), and user ratings may help in determining the areas where digital health apps can improve.

**Objective:**

This study has two objectives: (1) discover the types (clusters) of digital health apps with regards to their quality (scores) across 3 domains (their PCA, UX, and DP) and user ratings and (2) determine whether the National Institute for Health and Care Excellence (NICE) Evidence Standard Framework’s (ESF’s) tier, target users of the digital health apps, categories, or features have any association with this typology.

**Methods:**

Data were obtained from 1402 digital health app assessments conducted using the Organisation for the Review of Care and Health Apps Baseline Review (OBR), evaluating PCA, UX, and DP. K-medoids clustering identified app typologies, with the optimal number of clusters determined using the elbow method. The Shapiro-Wilk test assessed normality of user ratings and OBR scores. Nonparametric Wilcoxon rank sum tests compared cluster differences in these metrics. Post hoc analysis examined the distribution of NICE ESF tiers, target users, categories, and features across clusters, using Fisher exact test with Bonferroni correction. Effect sizes were calculated using Cohen *w*.

**Results:**

A total of four distinct app clusters emerged: (1) apps with poor user ratings (220/1402, 15.7%), (2) apps with poor PCA and DP scores (252/1402, 18%), (3) apps with poor PCA scores (415/1402, 29.6%), and (4) higher quality apps with high user ratings and OBR scores (515/1402, 36.7%). While some statistically significant associations were found between clusters and NICE ESF tiers (2/3), target users (0/14), categories (4/33), and features (6/19), all had small effect sizes (Cohen *w*<0.3). The strongest associations were for the “Service Signposting” feature (Cohen *w*=0.24) and NICE ESF tier B (Cohen *w*=0.19).

**Conclusions:**

The largest cluster comprised high-quality apps with strong user ratings and OBR scores (515/1402, 36.7%). A significant proportion (415/1402, 29.6%) performed poorly in PCA despite performing well in other domains. Notably, user ratings did not consistently align with PCA scores; some apps scored highly with users but poorly in PCA and DP. The 4-cluster typology underscores areas needing improvement, particularly PCA. Findings suggest limited association between the examined app characteristics and quality clusters, indicating a need for further investigation into what factors truly influence app quality.

## Introduction

Digital health apps allow for proactive rather than reactive health care and have the potential to take the pressure off health care providers. Furthermore, the integration of digital health apps into clinical practice was shown to reduce the clinician’s workload in some cases [1,2], as well as increase workload in others [3]. According to an umbrella review from 2023 [4], digital health apps for noncommunicable diseases are effective in improving health outcomes. For mental health apps, it was reported that advances in digital health are changing mental health care in multiple ways, such as making mental health care more accessible [5]. The public appears to have a positive attitude regarding the use of digital health apps. According to the Organisation for the Review of Care and Health Apps (ORCHA) website [6], “68% of people surveyed agreed, or strongly agreed, that to take pressure off our health care system, it is vital we all look at new ways to manage our health, including using high-quality health apps” [7]. A meta-ethnographic review from 2019 [8], with the aim to explore the public perception of digital health apps, found that users viewed digital health apps as useful complementary tools. However, there are still barriers regarding their use and quality that need to be resolved.

A study from 2020 [9] indicates that the evidence whereby digital health apps improve patient outcomes is scarce. Continued use of digital health apps (ie, user retention) after their installation is also a concern. A systematic review from 2020 [10] found a high dropout rate (47.8% when adjusting for publication bias) among digital health apps for depressive symptoms. A study from 2019 [11], focused on the “continued use” of mobile health apps, found that for users to continue using health apps, users must have a positive view of the app’s user experience (UX) and be persistent at achieving their health goals. The effectiveness of digital health apps in the management of various chronic diseases remains unclear [12]. There are also barriers regarding the apps’ integration into clinical practice, such as patient or provider support [13].

A mixed methods study from 2019 [14] found that users are concerned with the security and privacy of digital health apps. Furthermore, the current literature suggests that digital health apps could be improved regarding their quality [5,15-21]. The limitation of these studies is that they were often conducted using a small sample of data and the analyzed apps were for a specific category or health condition, making their findings category or condition specific.

In our study, collaborating with the ORCHA, we included digital health apps from 33 different categories allowing us to get a “big picture” of their performance regarding quality. ORCHA used their assessment tool called ORCHA Baseline Review (OBR) [22] to quality assess over 1400 digital health apps. The tool assesses digital health apps regarding their UX, data privacy (DP), and professional and clinical assurance (PCA). The scores in the 3 assessment areas are then combined into an overall ORCHA score out of 100. The aim of this study is to uncover similarities and differences in traits among digital health apps regarding characteristics related to their quality (as indicated by OBR’s PCA, UX, and DP scores) and user ratings. We accomplish this aim with two objectives: (1) to discover the types (clusters) of digital health apps with regards to their quality (scores) across three domains (their PCA, UX, and DP) and user ratings and (2) to determine whether the National Institute for Health and Care Excellence (NICE) Evidence Standard Framework’s (ESF) tier, target users of the digital health apps, categories, or features have any association with this typology.

Uncovering the similarities and differences in the traits of digital health apps via k-medoids cluster analysis can indicate areas where digital health apps can improve regarding their quality assessment and inform the state of digital health apps today. It also allows us to see how different categories of digital health apps are impacted by the same drawbacks. Similar work, using different methods, was done to explore the traits of mental health apps [21]. In this cross-sectional study, researchers examined 578 mental health apps regarding features that they offer and found that the most common features are psychoeducation, goal tracking, and mindfulness. The study also found that DP is not associated with user ratings and is weakly associated with the number of downloads. A study from 2019 [23] analyzed the most popular mental health apps with the aim to understand how their attributes relate to user ratings, app quality, and classification by the World Health Organization (WHO) health app classification framework [24]. However, due to the heterogeneity of the apps, they were unable to define a core set of features that would accurately assess app quality. In our study, as a post hoc analysis, we examined each cluster regarding each digital health app’s NICE ESF tier [25], target users, categories, and features. This is done to examine whether there are any other relationships between a cluster or type of app and its NICE ESF tier, category, target users, or features of a digital health app. This study extends our “work in progress” paper from 2023 [26], applying more rigor and additional analyses. In our previous work, we conducted an analysis of digital health apps’ quality across different health care categories [27].

## Methods

### The Dataset

ORCHA has conducted digital health apps’ assessment as part of their standard workflow resulting in the original dataset of 2127 digital health apps. The OBR version 6 evolved from earlier versions of the OBR during the height of the COVID-19 pandemic. Originally, version 6 was created as a more stringent version of the OBR so that ORCHA could recommend the most compliant digital health apps to members of the UK population with confidence. ORCHA tested version 6 on a selection of highly compliant digital health apps (as determined by previous versions of the OBR). This set of 30 digital health apps served as the pilot group, with the subsequent 2097 apps being assessed with ORCHA’s typical assessment approach of categorizing apps into categories, ordering by number of downloads, and assessing the most downloaded apps in each category, followed by the second, and so forth. The apps have been selected from the original 2127 app assessments by removing apps without user ratings, second assessments of the same app, apps that were not digital health apps, and taking the mean results of app assessments with both Android and iOS versions of the app. Resulting in the dataset of 1402 digital health apps that were used in this study (see Results section).

The dataset consists of digital health app assessments that were published between January 18, 2021, and January 6, 2022. All digital health apps were characterized using 14 target user groups (eg, adult, carer, etc), 33 categories (eg, allergy, blood, etc) and 19 features (eg, behavioral change techniques, condition management, etc), as well as to one of the three NICE ESF tiers [25]: (1) Tier A (n=9), “Digital health technologies intended to save costs or release staff time, no direct patient, health or care outcomes;” (2) Tier B (n=1018), “Digital health technologies for helping citizens and patients to manage their own health and wellness;” and Tier C (n=375), “Digital health technologies for treating and diagnosing medical conditions, or guiding care choices. Includes digital health technologies with direct health outcomes, and those that are likely to be regulated medical devices.” Details on how the digital health apps have been selected can be found in the Results section.

### Assessment With ORCHA Baseline Review

A digital health app with satisfactory PCA, UX, and DP could lead to greater digital health app adoption, confident app recommendations by clinicians, and prevent risks that could arise from using the app. OBR assesses these 3 aspects of digital health apps. This is because assessing PCA ensures that the app is evidence-based and reduces the risks of an app having harmful effects on users’ health. PCA assessment includes questions such as: “Is the developer or publisher registered with the Care Quality Commission (CQC)?” and “Is the app a medical device?” Assessing UX ensures that the app is useful, usable, and desirable by the user. UX assessment includes questions such as: “Is there a statement about user feedback during design/ development?” and “Is there any evidence of user involvement in testing?.” Assessing DP ensures that users' rights and data are protected and not exploited in any way (eg, financially). DP assessment includes questions such as: “Is there a Privacy Policy clearly available via the App/ Web App/ Website?” and “Is the policy made available when the user is signing up to the service?” Digital health apps in the dataset are assigned an ORCHA score (range 0‐100). ORCHA score is based on the 3 assessment areas (UX, PCA, and DP), each of which has their own score out of 100, where higher value indicates better compliance.

Each app was assessed by 2 ORCHA reviewers using OBR. In the case of a dispute, a third reviewer would be involved to resolve it. The reviewers assessed digital health apps using OBR assessment to get the following outcomes: PCA (indicated by PCA score), DP (indicated by DP score), UX (indicated by UX score), and an overall ORCHA score. In the dataset, 5-star user ratings at the app store were recorded at the time of assessment. An ORCHA threshold score of 65 out of 100 is a National Health Service (NHS) accepted cut-off point that indicates compliance with best practice standards for digital health apps, meaning that the digital health app may be used or recommended by NHS staff. ORCHA threshold is the point at which excess risks are avoided, that is, an app cannot possibly score above 65 while having no privacy policy, having no relevant evidence, or being a medical device which is not certified. The score of 65 was established with NHS partners in 2020 and has remained since.

An ORCHA score of 65 is an initial score for all the digital health apps being assessed in all assessment areas (UX, PCA, and DP). Meaning that the initial score at the beginning of the assessment is 65 for each assessment area and overall ORCHA score. Based on answers to assessment questions, this score is altered via value and risk points. Value points increase the score, and risk points reduce the score. This process changes the initial score of 65 for each assessment area and is then combined to give an overall ORCHA score. For example, for the apps that store personal and sensitive information, value points are assigned to such an app if they make their privacy policy immediately available when the user first uses the app. Risk points are assigned if a privacy policy is not clearly available via the app. The amount of value and risk points assigned per question varies based on the NICE ESF tier that was assigned to an app. If no value or risk points were assigned during the assessment, then the ORCHA score remains 65 [22]. Furthermore, to receive full points for appropriate evidence for its NICE ESF tier, a tier B digital health app (depending on its exact functionality) may only require a user benefits statement (eg, based on pilot results) and validation of the provided information by experts or references, while a tier C digital health app will likely require a full-scale observational study or randomized controlled trial to meet the same evidence threshold. These differences in evidence requirements were introduced by the NICE ESF and adopted with slight amendments by the ORCHA assessment. This was done to ensure that standards are realistic and achievable for digital health app companies, without placing an undue burden on developers of low-risk apps, while at the same time setting expectations sufficiently high for high-risk apps.

In summary, digital health apps with an ORCHA score of <65 are not recommended to be used by the public. Having a score <65 on an assessment area (UX, PCA, or DP) indicates that improvements should be made in that area by developers. However, it may still be recommended for use if other areas (especially PCA, due to its reliance on evidence) are >65.

### Statistical Analysis

R studio and the R programming language was used to conduct the analysis and produce the graphs. Spearman correlation among the OBR scores (ORCHA, PCA, UX, and DP) and user ratings was calculated to examine the relationship among variables. Median (IQR) was calculated for the OBR scores and user ratings for reference. The Shapiro-Wilk test was used to check if the OBR scores or user ratings were normally distributed. K-medoids clustering was used to group together (classify) health apps based on their user ratings and PCA, UX, and DP scores. Due to the scores not being normally distributed, K-medoids clustering was used to allow for a more fitting interpretation of clustering results, not skewed by outliers in the cluster. K-medoids clustering is a partitioning technique based on medoids. A medoid is the most centrally located point in a cluster, with the minimum sum of distances to other points. Clustering was used as it simplifies the grouping together (classification) of apps. We have chosen clustering to discover the types of apps because this approach is objectively data-driven and does not result in bias and error from manual grouping (classification). A widely used elbow method was used to determine the optimal number of clusters for the K-medoids cluster analysis. Following the results of the Shapiro-Wilk tests (indicating that user ratings and PCA, UX, and DP scores are not normally distributed), the unpaired 2-samples Wilcoxon rank sum test (also known as Mann-Whitney *U* test) was used to compare the corresponding user ratings and the scores among clusters, to check for statistical significance. The Wilcoxon test was used due to the data not being normally distributed. A *P* value less than .05 was considered statistically significant. Bonferroni corrected alpha value was used when multiple hypothesis testing was conducted on the same data.

The following steps have been taken when conducting the analysis:

- Step 1, outcomes under investigation: digital health apps’ user ratings, PCA, UX, and DP scores will be investigated from the assessment data in the dataset. Those values were obtained by assessing digital health apps with OBR.
- Step 2, outcomes assessment: K-medoids clustering will be used on the user ratings, PCA, UX, and DP scores from the dataset.
- Step 3, outcomes interpretation: each cluster will have a medoid value where user ratings range from 1 to 5, where higher numbers indicate better rating. PCA, UX, and DP scores range from 0 to 100 where higher numbers indicate better compliance with assessment area.
- Step 4, variables generated: clusters will be investigated and labeled based on the medoid value of user ratings, PCA, UX, and DP scores in each of the clusters.

Post hoc analysis will be conducted by counting the prevalence of each target user group, categories, and features in each cluster. Fisher exact test will be used to determine whether the difference in proportion was statistically significant among the clusters. When the Fisher exact test has a statistically significant Bonferroni corrected alpha, the effect size will be calculated using Cohen *w*. A widely used “rule of thumb” interpretation of Cohen *w* for the effect size is as follows: small (Cohen *w*=.10), medium (Cohen *w*=.30), and large (Cohen *w*=.50) [28]. However, this interpretation was criticized, and a new interpretation was proposed by Funder and Ozer [29]: very small (Cohen *w*=.05), small (Cohen *w*=.10), medium (Cohen *w*=.20), large (Cohen *w*=.30), and very large (Cohen *w*≥.40). Both interpretations will be considered in this study.

### Ethical Considerations

This secondary data analysis study gained ethical approval by Ulster University (ethics filter committee, Faculty of Computing, Engineering, and the Built Environment; project CEBE_RE-22-002). The data used in this study has been anonymized. The developers under consideration provided implicit consent for use of their data for research purposes. All reviews, unless explicitly asked to be removed by the developer, are covered as suitable for research in ORCHA’s privacy policy [30].

## Results

### Principal Results

**Figure 1.**
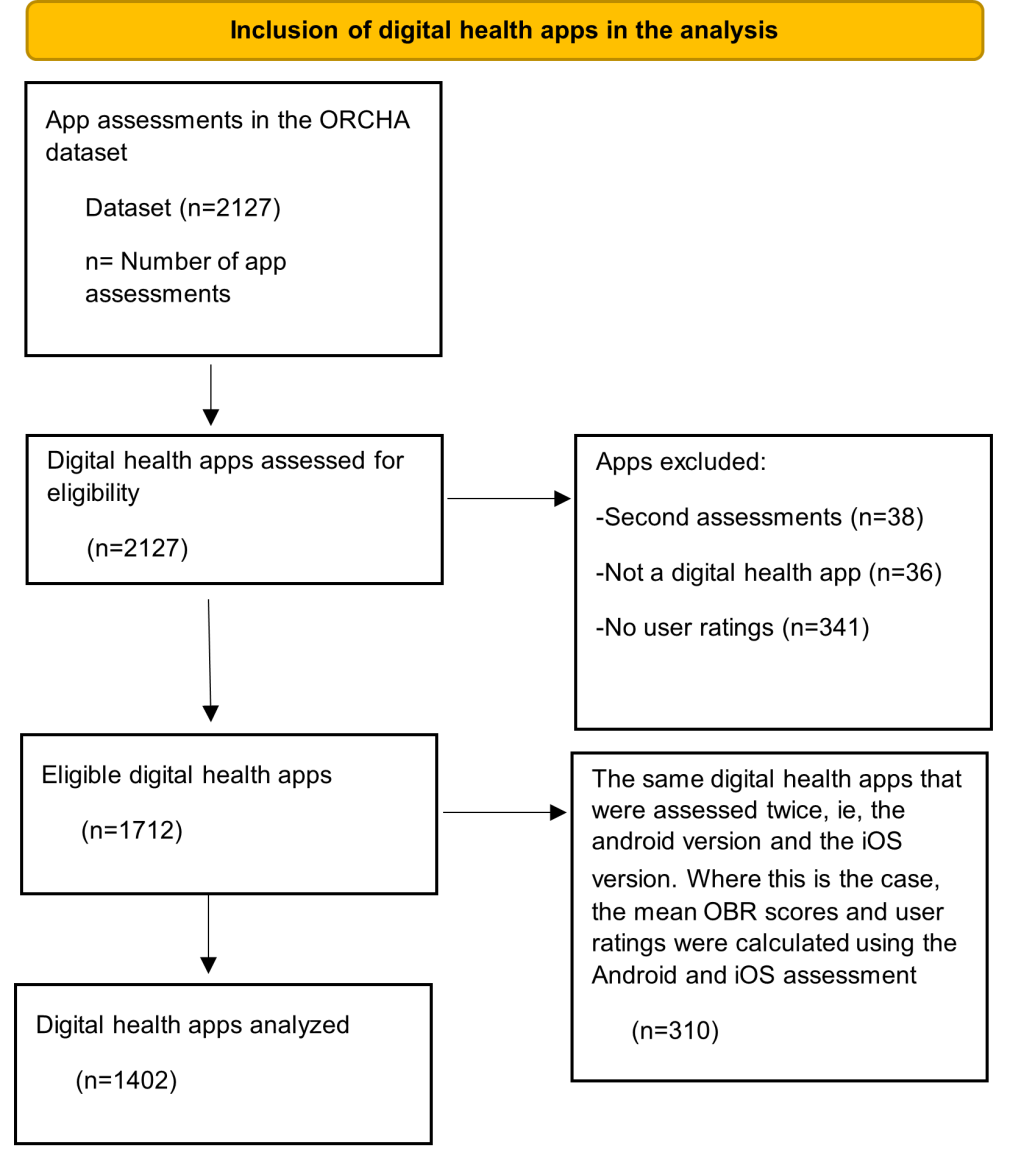
Digital health apps selection diagram. ORCHA: Organisation for the Review of Care and Health Apps; OBR: ORCHA Baseline Review.

Figure 1 shows how the 1402 digital health apps have been selected from the original 2127 digital health apps assessed by ORCHA. To avoid inclusion of the same app twice in the analysis, the mean of the scores (ORCHA, PCA, UX, and DP) and user ratings was taken from the 2 versions (iOS and Android) and included in the analysis as one digital health app.

Table 1 depicts Spearman correlations and *P* values among the OBR scores (ORCHA, PCA, UX, and DP) and user ratings. All the OBR scores obtained a *P* value of <.001 when correlated with other OBR scores. User ratings did not obtain statistically significant correlations with any of the OBR scores. Shapiro-Wilk test results were <.001 for all the OBR scores and user ratings. Indicating that the variables are not normally distributed. Table 1 shows that achieving a high score in one dimension of PCA, UX, and DP is not a good predictor for the other two dimensions. However, PCA score is a good predictor of an overall ORCHA score, as can be seen by high Spearman correlation of .940.

**Figure 2.**
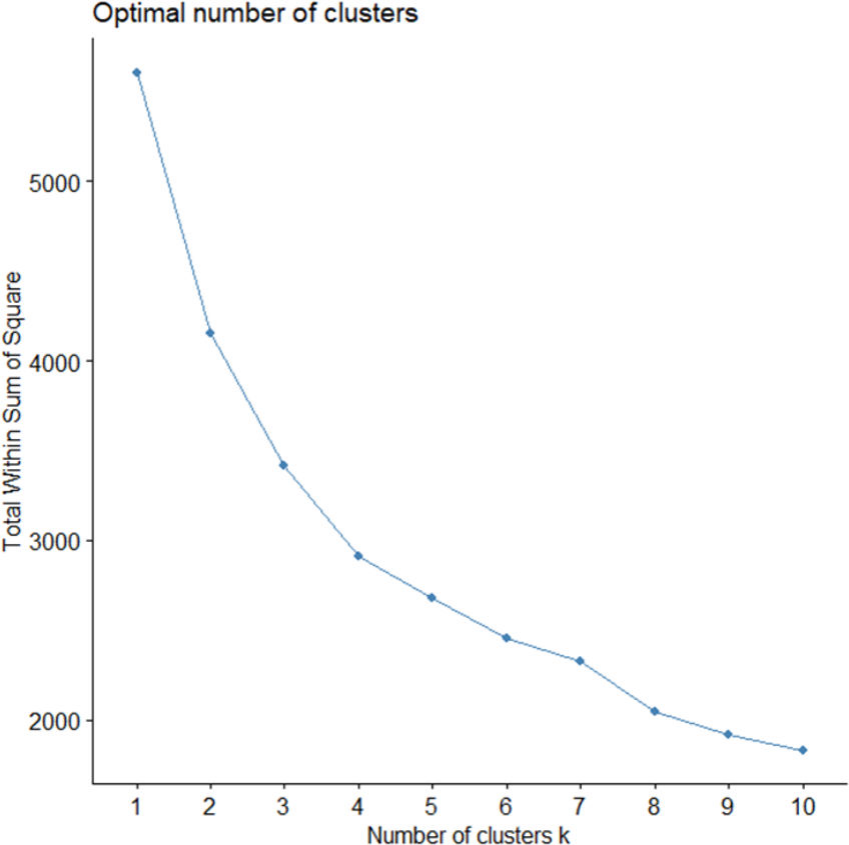
Optimal cluster selection—elbow method.

**Figure 3.**
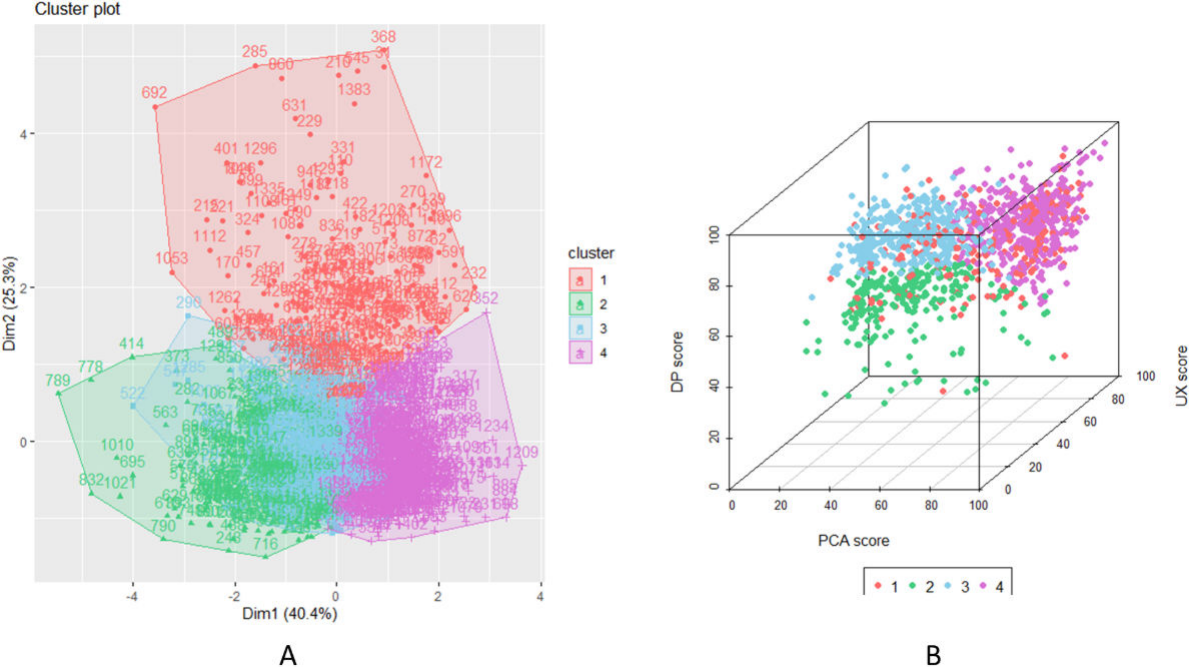
(A) K-medoids cluster plot and (B) 3D plot of user experience (UX), professional and clinical assurance (PCA), and data privacy (DP) separated by clusters.

K-medoids clustering was performed on the sample size (n) of 1402 digital health apps regarding their quality scores (UX, PCA, and DP) on the ORCHA assessment tool and user ratings. K-medoids had been used due to being less sensitive algorithm to outliers in data not assuming all variable to have the same variance. Figure 2 shows the results of the elbow method that was used to determine optimal number of clusters for the analysis. Considering the Elbow method, 4 clusters have been determined as the most optimal; see Multimedia Appendix 1 for analysis with 3, 5, and 6 clusters. Figure 3 depicts a k-medoids cluster plot of 4 clusters that were used to assign digital health apps based on their quality scores (UX, PCA, and DP) and their app store user ratings.

Each of the 4 clusters used for the analysis was assigned a label that best describes them. Table 2 depicts cluster number, label, and description. The labels indicate where improvements can be made, except for cluster 4 labeled “Higher quality apps with higher user ratings,” as this cluster represents ideal (regarding quality) digital health apps. Table 3 depicts the median (IQR) of each variable used in the k-medoids clustering and cluster medoids for each of the four clusters, as well as ORCHA score median (IQR) and the cluster size.

**Figure 4.**
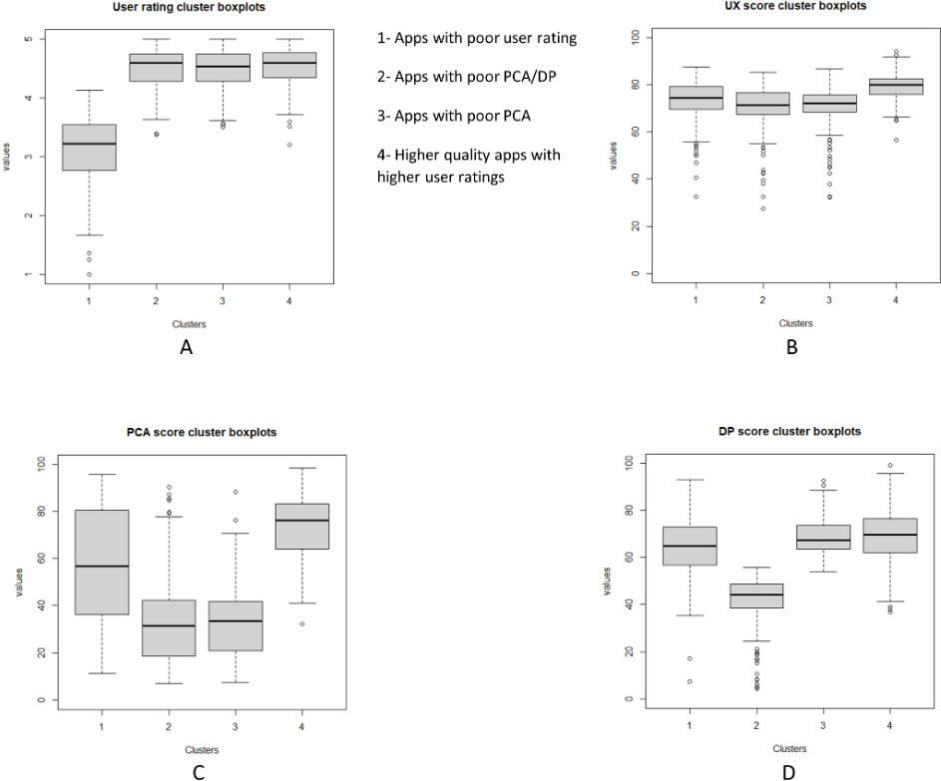
Boxplot of user ratings and Organisation for the Review of Care and Health Applications. Baseline Review (OBR) scores per cluster. (A) user ratings cluster boxplots, (B) user experience (UX) cluster boxplots, (C) professional and clinical assurance (PCA) cluster boxplots, and (D) data privacy (DP) cluster boxplots.

Figure 4 shows the value of each variable used in clustering per cluster in boxplots. The labels for each of the variables used in clustering were determined based on values in Table 3 and on depictions in Figure 4 boxplots. Figure 4A depicts user rating boxplots that show that user ratings are high for all clusters except for the cluster number 1 “Apps with poor user ratings.” Figure 4B depicts UX score boxplots that show that UX is high for all clusters. In Figure 4C, PCA score boxplots show that PCA is high for cluster number 4 “Higher quality apps with higher user ratings,” variable for cluster number 1 “Apps with poor user ratings,” and low for 2 clusters “Apps with poor PCA/DP” and “Apps with poor PCA.” Finally, Figure 4D shows DP score boxplots that show that DP is high for 3 clusters and lower for cluster number 2 “Apps with poor PCA/DP.” Multimedia Appendix 2 shows Wilcoxon rank sum test for each of the variables against each cluster.

K-medoids clustering conducted with 3, 5, and 6 clusters can be found in Multimedia Appendix 1. Clustering results with 3 clusters indicated that further clustering may be possible, whereas clustering with 5 and 6 clusters indicated that the number can be reduced. For clustering with 5 clusters, cluster number 5 has intermediate results for PCA and DP scores, indicating that digital health apps in this cluster may be split into clusters with higher and lower PCA and DP scores. For clustering with 6 clusters, 2 of the same labels can be used to describe 4 (out of 6) different clusters, further indicating that the number of clusters should be lower. Hence, for the rest of the analyses, results with 4 clusters have been used.

**Table 1.** ORCHA Baseline Review (OBR) scores and user ratings Spearman correlations and *P* values.

Variables		Correlation	*P* value
ORCHA^a^ score			
	User ratings	.002	..94
	PCA^b^ score	.94	<.001
	UX^c^ score	.53	<.001
	DP^d^ score	.57	<.001
PCA score			
	User ratings	−.02	.57
	UX score	.41	<.001
	DP score	.33	<.001
UX score			
	User ratings	.04	.14
	DP score	.23	<.001
DP score			
	User ratings	−.02	.37

a ORCHA: Organisation for the Review of Care and Health Apps.

b PCA: professional and clinical assurance.

c UX: user experience.

d DP: data privacy.

**Table 2.** Cluster number, label, and description.

Cluster number	Cluster label	Description	Cluster size, n (%)
1	Apps with poor user ratings	These are the apps that have low user ratings, but have intermediate DP^a^, PCA^b^ scores, and a high UX^c^ score.	220 (15.7)
2	Apps with poor PCA and DP	These are the apps with low PCA and DP scores but have high UX scores and user ratings.	252 (18)
3	Apps with poor PCA	These are the apps with a low PCA score but high UX and DP scores and high user ratings.	415 (29.6)
4	Higher quality apps with higher user ratings	These are apps with high user ratings and high scores across all 3 domains.	515 (36.7)

a DP: data privacy.

b PCA: professional and clinical assurance.

c UX: user experience.

**Table 3.** Cluster centers of 1402 digital health apps, with cluster size percentage (%) out of 1402.

Variables	Median (IQR)	Cluster medoids			
		1	2	3	4
		Apps with poor user ratings	Apps with poor PCA^a^ and DP^b^ scores	Apps with poor PCA scores	Higher quality apps with higher user ratings
User ratings	4.49 (0.707)	3.23	4.58	4.47	4.60
PCA score	49 (45.3)	57.0	35.1	31.9	73.0
UX^c^ score	75.2 (9.42)	72.9	71.6	71.6	80.3
DP score	65.3 (18.3)	61.3	42.4	67.5	66.4
ORCHA^d^ score, median (IQR)	—^e^	63 (21)	46 (12)	54 (9)	74 (10)
Cluster size, n (%)	—	220 (15.7)	252 (18)	415 (29.6)	515 (36.7)

a PCA: professional and clinical assurance.

b DP: data privacy.

c UX: user experience.

d ORCHA: Organisation for the Review of Care and Health Applications.

e Not applicable.

### Post Hoc Analysis

Fisher exact test with Bonferroni corrected alpha value was conducted between smallest and largest cluster percentage for NICE ESF tiers, target users, categories, and features. Statistical significance with Bonferroni corrected alpha value was found in 2/3 NICE ESF tiers, 0/14 target users, 4/33 categories, and 6/19 features. Results are presented in Multimedia Appendix 2 and Multimedia Appendix 3.

## Discussion

### Principal Findings

K-medoids clustering analysis was conducted using 4 clusters. The elbow method was used to select the optimal number of clusters. Different clusters were also tried; see Multimedia Appendix 1 for analysis with 3, 5, and 6 clusters. The clustering was performed on 4 variables: user ratings, PCA score, UX score, and DP score (see Table 3 and Figure 4). The four clusters were labeled: (1) apps with poor user ratings (n=220), (2) apps with poor PCA and DP scores (n=252), (3) apps with poor PCA scores (n=415), and (4) higher quality apps with higher user ratings (n=515). See Table 2 for label description. Considering the OBR scores, user ratings, and cluster size, the following conclusions were drawn:

The most common digital health app (in this dataset) is those with high user ratings and high OBR quality scores, as indicated by the cluster labeled “higher quality apps with higher user ratings,” n=515 (36.7%).

There are many digital health apps that lack PCA but excel in user ratings, UX, and DP scores, as indicated by the cluster “Apps with poor PCA scores,” n=415 (29.6%). Meaning these apps may be portrayed as good quality apps, despite not being evidence-based.

User ratings are not necessarily indicative of OBR quality assessment scoring. A digital health app can receive high user ratings and low OBR scores and vice versa, as indicated by Table 1. The finding that user ratings are not linked to OBR scores was confirmed with different analysis using the subset of the same original dataset [31].

As revealed by Hyzy et al [31], widely available proxies that users may perceive to signify the quality of health apps, namely user ratings and downloads, were found to be inaccurate predictors for estimating quality. That study indicated the need for the wider use of quality assurance methodologies that can accurately determine the quality, safety, and compliance of health apps, and this research clearly confirms that finding indicating that more should be done to enable users to recognize high-quality health apps, including digital health literacy training and the provision of nationally endorsed “libraries.”

The discovered typology could be used by assessors to classify future apps. The typology can also be used to track the size of these clusters over time. Looking for changes and trends—a kind of digital health app surveillance.

To further understand the clusters, a post hoc analysis was conducted, examining the spread of NICE ESF tiers, target users, categories, and features across the 4 clusters. Multimedia Appendix 3 shows the assigned NICE ESF tiers for each cluster. Multimedia Appendix 4 shows assigned target users, categories, and features for each of the clusters. This was done to check whether there is any relationship among NICE ESF tiers, target users, categories and features, and the clusters.

Fisher exact test with Bonferroni corrected alpha value was used between the largest and the smallest percentage of prevalence of NICE ESF tiers, target users, categories, and features relative to the cluster size. Statistical significance with Bonferroni corrected alpha value was achieved by 2/3 NICE ESF tiers, 0/14 target users, 4/33 categories, and 6/19 features. Results are presented in Multimedia Appendix 2 and Multimedia Appendix 3. For example, for the category “Ophthalmology,” the cluster labeled “Higher quality apps with higher user ratings” had the smallest percentage of prevalence of .583% and the cluster labeled “Apps with poor PCA/DP” had the largest percentage of prevalence of 6.75%, relative to cluster size. The Fisher exact test *P* value for the 2 clusters was <.001, with Bonferroni corrected alpha value of .002. These results are indicating statistically significant differences between the clusters with a Cohen *w* of .182. This means that there is a statistically significant association between some NICE ESF tiers, target users, categories, and features, and the clusters. However, the effect size was small, Cohen *w*<.2, for most NICE ESF tiers, target users, categories, and features that achieved statistical significance. Most noteworthy was the NICE ESF tier B and C with Cohen *w* of .193 and .190, respectively. As well as the category “Ophthalmology” and the app feature “Service Signposting” with Cohen *w* of .182 and .241, respectively, indicating small-medium effect size when considering both Cohen *w*s [28] and Funder and Ozer [29] interpretation. Hence, the results seem to indicate that NICE ESF tiers, target user, categories, and features, by and large, do not have an effect on the typology of digital health apps.

Previous studies have been done examining quality and impact of digital health apps, as well as finding areas for improvement. A review from 2022 [32] examined the impact of digital health apps. The review discussed that there is little evidence to show that digital health apps impact health outcomes either positively or negatively. However, a study from 2021 [33] discussed the impact of telemedicine and the use of digital health apps for healthcare. The study found that the use of telemedicine and digital health apps can streamline the workflow of hospitals and clinics; for example, scheduling follow-up visits may allow doctors and patients to be more effective and optimize patient outcomes. Hence, digital health apps can be a good complementary tool to standard health care. A review from 2023 [34] discussed barriers to the use of digital health apps. The review revealed 10 major barriers and problems associated with the use of digital health apps. Those were “validity,” “usability,” “technology,” “use and adherence,” “data privacy and data security,” “patient-physician relationship,” “knowledge and skills,” “individuality,” “implementation,” and “costs.” Furthermore, the results showed that more research is needed to study the problems and barriers of digital health apps. A review from 2021 [35] examined the effectiveness of digital health apps to promote health and manage disease. The review found a steady increase in the rigorous assessment of digital health apps. Although it also found that there is a need for improved methodological and assessment approaches. Our study of categorizing digital health apps into a 4-cluster typology could lead to improving the quality of digital health apps, as it identifies areas of improvement and how prevalent they are.

Further analyses could be conducted on OBR scores and NICE ESF tiers, target user, categories, and app features. To examine how the OBR score changed when partitioned by NICE ESF tiers, target users, and categories. Or whether having specific features is linked to higher OBR scores. Furthermore, studies can be done to attempt to find the underlying cause for the 4-cluster typology. Possible analyses include whether the inclusion of a clinician during app development improves the app’s quality. Or does having a higher budget during app development lead to higher quality of apps?

Further analysis could be done to reproduce this study using different assessment frameworks. The study conducted in this paper was done using the ORCHA assessment tool called OBR. However, there are alternative frameworks that could be used to assess apps, such as the Mobile App Rating Scale (MARS) [36]. OBR and MARS also use different rating techniques, where OBR uses mostly polar yes or no questions assigning risk and value points, whereas MARS uses 5-point scale questions. Another alternative is the Enlight [37] framework, which uses 5-point scale questions and checklists.

It may be helpful if app stores, or app developers, presented PCA of digital health apps in a standardized way akin to a privacy policy. Perhaps users can view the underpinning evidence of the app or content in the app itself and see answers to basic questions such as “how many healthcare professionals reviewed or were involved in the design of the app?” This would allow users to make more informed decisions on whether to use an app or not.

### Limitations

This analysis was conducted using 4 clusters, and using a different number of clusters may have led to slightly different results. However, the elbow method has been used to select the number of clusters, and using different number of clusters in Multimedia Appendix 1 indicated that 4 clusters is most suitable. Using a different clustering solution such as k-means, self-organizing maps (SOMs), etc, may have also led to different results. Initially, we considered using k-means clustering but changed to k-medoids due to the presence of outliers in the data. When conducting post hoc analysis on target users, categories, and features, only the lowest and highest relative percentage (to cluster size) of prevalence have been used in Fisher exact test. When partitioning the clusters by NICE ESF tiers, target users, categories, and features, sample sizes have been low for some, making results less reliable. A dataset with a larger sample size could lead to more accurate results. Furthermore, the dataset may not be representative of all health apps since it is based on apps that were submitted to ORCHA for assessment. Meaning the dataset does not include apps that were not submitted to be assessed. Hence, the dataset is not a random sample of health apps and there may be a sample bias. As a result, a random sample of health apps might result in different results and clusters.

This analysis was done on the assessment conducted using the OBR assessment tool, using a different tool (eg, MARS [36] or Enlight [37]) could lead to different results. Using a quality assessment tool designed to assess a specific category of a digital health app could lead to more accurate results (category-specific typology) that could be used to improve their quality. Analysis of the OBR assessment questions for each of the clusters could have explained or indicated what led to the 4-cluster typology.

### Conclusion

This study, based on the OBR assessments of 1402 digital health apps, shows that digital health apps can be assigned into a typology of 4 clusters (based on k-medoids clustering). The clusters were labeled: (1) apps with poor user ratings (220/1402, 15.7%), (2) apps with poor PCA and DP scores (252/1402, 18.0%), (3) apps with poor PCA scores (415/1402, 29.6%), and (4) higher quality apps with higher user ratings (515/1402, 36.7%). The principal findings of the analysis were: (1) the most common digital health apps are those with high user ratings and with high OBR scores indicating that these apps are of high quality and are well received by users (515/1402, 36.7%); (2) there are many digital health apps that lack PCA yet excel in user ratings, UX, and DP scores (415/1402, 29.6%), which may be dangerous as it means that many digital health apps are not evidence-based, but due to high user ratings, as well as good UX and DP policy, they may be portrayed as good quality apps; and (3) user ratings are not necessarily indicative of quality (according to OBR assessment scores), as digital health apps can receive high user ratings and low OBR scores and vice versa. Furthermore, for some digital health apps, NICE ESF tier and, to a lesser extent, category and features had a statistically significant effect on the assigned cluster. However, effect size with Cohen *w* was <.3 for all, indicating small association. This study indicates that many digital health apps could be improved regarding either their PCA or DP (667/1402, 47.6%). Also, 15.7% (220/1402) of digital health apps were of good quality but received poorer user ratings than other clusters; this might be due to apps not meeting user demands but providing accurate research-informed content. Further research is needed to understand the underlying cause for the 4-cluster typology of digital health apps.

Knowledge of the quality shortcomings in digital health apps and how prevalent they are as shown by the 4 clusters and their cluster size can inform the direction needed for future research. This study showed that the examined NICE ESF tiers, target users, categories, and features of digital health apps are not strongly associated with the 4-cluster typology of digital health apps.

## Supplementary material

10.2196/57279 Multimedia Appendix 1 Digital health apps clusters.

10.2196/57279 Multimedia Appendix 2 Digital health apps cluster values comparison (4-clusters typology).

10.2196/57279 Multimedia Appendix 3 Clusters per National Institute for Health and Care Excellence Evidence Standard Framework (NICE ESF) tier.

10.2196/57279 Multimedia Appendix 4 Digital health apps’ target user, categories, and features per cluster.

## Abbreviations

DP: data privacy
ESF: Evidence Standard Framework
MARS: Mobile App Rating Scale
MAUQ: mHealth App Usability Questionnaire
NHS: National Health Service
NICE: National Institute for Health and Care Excellence
OBR: Organisation for the Review of Care and Health Applications Baseline Review
ORCHA: Organisation for the Review of Care and Health Applications
PCA: professional and clinical assurance
UX: user experience
WHO: World Health Organization
