# Supplementary material for: Grouping Digital Health Apps Based on Their Quality and User Ratings Using K-Medoids Clustering: Cross-Sectional Study
Source: JMIR Mhealth Uhealth. 2025 Jul 23;13:e57279. doi: 10.2196/57279 (PMC12309620; doi:10.2196/57279)
Supplement: Multimedia Appendix 1 [file mhealth-v13-e57279-s001.docx]

## Appendix 1 – Digital health apps clusters

The following tables depict K-medoids clustering done with 3, 5 and 6 clusters.

***Appendix 1 Table 1****: K-medoids clustering with 3 clusters.*

|  |  | **Cluster medoids** | | |
| --- | --- | --- | --- | --- |
|  |  | 1 | 2 | 3 |
| **Variables** | **Median (IQR)** | Apps with poorer user ratings | Apps with poor PCA/DP scores | Higher quality apps with higher user ratings |
| User rating | 4.49(.707) | 3.46 | 4.56 | 4.72 |
| PCA score | 49.0(45.3) | 47.7 | 29.4 | 69.8 |
| UX score | 75.2(9.42) | 76.7 | 70.0 | 78.9 |
| DP score | 65.3(18.3) | 68.2 | 53.9 | 69.3 |
|  | **Median ORCHA score (IQR)** | 62(17.5) | 49(10) | 73(11) |
|  | **Cluster size (%)** | 295 (21.0%) | 525(37.4%) | 582(41.5%) |

***Appendix 1 Table 2****: K-medoids clustering with 5 clusters.*

|  |  | **Cluster medoids** | | | | |
| --- | --- | --- | --- | --- | --- | --- |
|  |  | 1 | 2 | 3 | 4 | 5 |
| **Variables** | **Median (IQR)** | Apps with poor user ratings | Apps with poor PCA/DP scores | Apps with poor PCA scores | Higher quality apps with higher user ratings | Apps with intermediate PCA/DP |
| User rating | 4.49(.707) | 2.92 | 4.58 | 4.47 | 4.53 | 4.61 |
| PCA score | 49.0(45.3) | 55.0 | 35.1 | 31.9 | 79.3 | 63.6 |
| UX score | 75.2(9.42) | 75.2 | 71.6 | 71.6 | 77.5 | 79.6 |
| DP score | 65.3(18.3) | 60.8 | 42.4 | 67.5 | 74.2 | 60.1 |
|  | **Median ORCHA score (IQR)** | 62(20) | 46(9.88) | 54(9) | 77(7) | 67(7.75) |
|  | **Cluster size (%)** | 175(12.5%) | 230(16.4%) | 401(28.6%) | 333(23.8%) | 263(18.8%) |

***Appendix 1 Table 3****: K-medoids clustering with 6 clusters.*

|  |  | **Cluster medoids** | | | | | | |
| --- | --- | --- | --- | --- | --- | --- | --- | --- |
|  |  | 1 | 2 | 3 | 4 | 5 | 6 |  |
| **Variables** | **Median (IQR)** | Apps with poor user ratings | Apps with poor PCA/DP scores | Apps with poor PCA scores | Higher quality apps with higher user ratings | Higher quality apps with higher user ratings | Apps with poor PCA/DP scores |  |
| User rating | 4.49(.707) | 2.92 | 4.52 | 4.47 | 4.51 | 4.44 | 4.62 |  |
| PCA score | 49.0(45.3) | 55.02 | 22.41 | 31.88 | 74.54 | 77.72 | 50.89 |  |
| UX score | 75.2(9.42) | 75.2 | 68.7 | 71.6 | 82.5 | 73.0 | 78.1 |  |
| DP score | 65.3(18.3) | 60.8 | 45.7 | 67.5 | 72.8 | 70.4 | 50.3 |  |
|  | **Median ORCHA score (IQR)** | 62(20) | 42(7.75) | 54(9) | 75.8(9) | 75(7) | 60(12) |  |
|  | **Cluster size (%)** | 167(11.9%) | 190(13.6%) | 367(26.2%) | 292(20.8%) | 204(14.6%) | 182(13.0%) |  |
