# Supplementary material for: Grouping Digital Health Apps Based on Their Quality and User Ratings Using K-Medoids Clustering: Cross-Sectional Study
Source: JMIR Mhealth Uhealth. 2025 Jul 23;13:e57279. doi: 10.2196/57279 (PMC12309620; doi:10.2196/57279)
Supplement: Multimedia Appendix 2 [file mhealth-v13-e57279-s002.docx]

## Appendix 2 – Digital health apps cluster values comparison

**Appendix 2 Table 1:** Unpaired two-samples Wilcoxon test for user ratings, *p*-values. Bonferroni corrected alpha value .05/6 ≈ .008.

|  | Apps with poor user rating | Apps with poor PCA/DP | Apps with poor PCA | Higher quality apps with higher user ratings |
| --- | --- | --- | --- | --- |
| Apps with poor user rating |  |  |  |  |
| Apps with poor PCA/DP | <.001 |  |  |  |
| Apps with poor PCA | <.001 | .268 |  |  |
| Higher quality apps with higher user ratings | <.001 | .365 | .019 |  |

**Appendix 2 Table 2:** Unpaired two-samples Wilcoxon test for PCA scores, *p*-values. Bonferroni corrected alpha value .05/6 ≈ .008.

|  | Apps with poor user rating | Apps with poor PCA/DP | Apps with poor PCA | Higher quality apps with higher user ratings |
| --- | --- | --- | --- | --- |
| Apps with poor user rating |  |  |  |  |
| Apps with poor PCA/DP | <.001 |  |  |  |
| Apps with poor PCA | <.001 | .551 |  |  |
| Higher quality apps with higher user ratings | <.001 | <.001 | <.001 |  |

**Appendix 2 Table 3:** Unpaired two-samples Wilcoxon test for UX scores, *p*-values. Bonferroni corrected alpha value .05/6 ≈ .008.

|  | Apps with poor user rating | Apps with poor PCA/DP | Apps with poor PCA | Higher quality apps with higher user ratings |
| --- | --- | --- | --- | --- |
| Apps with poor user rating |  |  |  |  |
| Apps with poor PCA/DP | <.001 |  |  |  |
| Apps with poor PCA | <.001 | .678 |  |  |
| Higher quality apps with higher user ratings | <.001 | <.001 | <.001 |  |

**Appendix 2 Table 4:** Unpaired two-samples Wilcoxon test for DP scores, *p*-values. Bonferroni corrected alpha value .05/6 ≈ .008.

|  | Apps with poor user rating | Apps with poor PCA/DP | Apps with poor PCA | Higher quality apps with higher user ratings |
| --- | --- | --- | --- | --- |
| Apps with poor user rating |  |  |  |  |
| Apps with poor PCA/DP | <.001 |  |  |  |
| Apps with poor PCA | <.001 | <.001 |  |  |
| Higher quality apps with higher user ratings | <.001 | <.001 | .019 |  |
