# Supplementary material for: Grouping Digital Health Apps Based on Their Quality and User Ratings Using K-Medoids Clustering: Cross-Sectional Study
Source: JMIR Mhealth Uhealth. 2025 Jul 23;13:e57279. doi: 10.2196/57279 (PMC12309620; doi:10.2196/57279)
Supplement: Multimedia Appendix 3 [file mhealth-v13-e57279-s003.docx]

## Appendix 3 – Clusters per NICE ESF tier

Cohen W has been calculated only when Fisher exact test *p*-value was lower than Bonferroni corrected alpha value.

**Appendix 3 Table 1**: NICE ESF tiers per cluster, NICE ESF tier assignment is mutually exclusive. Percentages (%) are of NICE ESF tier per cluster sample size. Bonferroni corrected alpha .05/3 ≈ .017.

|  | **Cluster** | | | |  |  |  | |
| --- | --- | --- | --- | --- | --- | --- | --- | --- |
| **NICE ESF Tier** | Apps with poor user rating  (n=220) | Apps with poor PCA/DP  (n=252) | Apps with poor PCA  (n=415) | Higher quality apps with higher user ratings  (n=515) | ***p*-value*** | **Cohen W** | **Total** |  |
| A | 4(1.82%) | 0(0%) | 3(.723%) | 2(.388%) | .047 | NA | 9(.642%) |  |
| B | 159(72.3%) | 181(71.8%) | 263(63.4%) | 415(80.6%) | <.001 | .193 | 1018(72.6%) |  |
| C | 57(25.9%) | 71(28.2%) | 149(35.9%) | 98(19.0%) | <.001 | .190 | 375(26.7%) |  |

*Fisher exact test *p*-value between largest (red) and smallest (yellow) relative cluster percentage.
