# Supplementary material for: Grouping Digital Health Apps Based on Their Quality and User Ratings Using K-Medoids Clustering: Cross-Sectional Study
Source: JMIR Mhealth Uhealth. 2025 Jul 23;13:e57279. doi: 10.2196/57279 (PMC12309620; doi:10.2196/57279)
Supplement: Multimedia Appendix 4 [file mhealth-v13-e57279-s004.docx]

## Appendix 4 – Digital health apps’ target user, categories, and features per cluster

Cohen W has been calculated only when Fisher exact test *p*-value was lower than Bonferroni corrected alpha value.

**Appendix 4 Table 1**: Target user per cluster, target user assignment is mutually inclusive. Percentages (%) are of target user per cluster sample size.
Bonferroni corrected alpha .05/14 ≈ .004.

|  | **Cluster** | | | |  |  |  |
| --- | --- | --- | --- | --- | --- | --- | --- |
| **Target user** | Apps with poor user rating  (n=220) | Apps with poor PCA/DP  (n=252) | Apps with poor PCA  (n=415) | Higher quality apps with higher user ratings  (n=515) | ***p*-value*** | **Cohen W** | **Total** |
| Adult | 99(45.0%) | 122(48.4%) | 204(49.2%) | 265(51.5%) | .126 | NA | 690 |
| Carer | 4(1.82%) | 1(.397%) | 2(.482%) | 7(1.36%) | .189 | NA | 14 |
| Child | 15(6.82%) | 13(5.16%) | 21(5.06%) | 33(6.41%) | .371 | NA | 82 |
| Clinicians | 18(8.18%) | 14(5.56%) | 27(6.51%) | 43(8.35%) | .189 | NA | 102 |
| Everyone | 100(45.5%) | 112(44.4%) | 186(44.8%) | 203(39.4%) | .141 | NA | 601 |
| Healthcare administrator | 0(0%) | 1(.397%) | 0(0%) | 0(0%) | .329 | NA | 1 |
| Hearing impairments | 0(0%) | 0(0%) | 2(.482%) | 0(0%) | .199 | NA | 2 |
| Infant | 0(0%) | 1(.397%) | 0(0%) | 2(.388%) | .378 | NA | 3 |
| Patients | 8(3.64%) | 2(.794%) | 2(.482%) | 10(1.94%) | .004 | NA | 22 |
| Pre-teen | 5(2.27%) | 6(2.38%) | 12(2.89%) | 18(3.50%) | .491 | NA | 41 |
| Research participants | 1(.455%) | 0(0%) | 1(.241%) | 1(.194%) | .466 | NA | 3 |
| Teen | 37(16.8%) | 62(24.6%) | 91(21.9%) | 123(23.9%) | .042 | NA | 313 |
| Elderly/older adults | 23(10.5%) | 31(12.3%) | 47(11.3%) | 54(10.5%) | .564 | NA | 155 |
| Visual impairments | 1(.455%) | 4(1.59%) | 3(.723%) | 1(.194%) | .042 | NA | 9 |

*Fisher exact test *p*-value between largest (red) and smallest (yellow) relative cluster percentage.

|  | **Cluster** | | | | |  | |  | |  | |
| --- | --- | --- | --- | --- | --- | --- | --- | --- | --- | --- | --- |
| **Category** | Apps with poor user rating  (n=220) | Apps with poor PCA/DP  (n=252) | Apps with poor PCA  (n=415) | Higher quality apps with higher user ratings  (n=515) | ***p*-value*** | | **Cohen W** | | **Total** | |  |
| Allergy | 1(.455%) | 6(2.38%) | 2(.482%) | 5(.971%) | .128 | | NA | | 14 | |  |
| Blood | 1(.455%) | 0(0%) | 0(0%) | 0(0%) | .299 | | NA | | 1 | |  |
| Cancer | 11(5.00%) | 4(1.59%) | 7(1.69%) | 22(4.27%) | .062 | | NA | | 44 | |  |
| Cardiology | 8(3.64%) | 3(1.19%) | 7(1.69%) | 11(2.14%) | .124 | | NA | | 29 | |  |
| Caring for elderly | 2(.909%) | 1(.397%) | 2(.482%) | 3(.583%) | .601 | | NA | | 8 | |  |
| Children’s health | 9(4.09%) | 4(1.59%) | 14(3.37%) | 31(6.02%) | .005 | | NA | | 58 | |  |
| Dental | 2(.909%) | 8(3.17%) | 5(1.20%) | 7(1.36%) | .114 | | NA | | 22 | |  |
| Diabetes | 17(7.73%) | 11(4.37%) | 14(3.37%) | 29(5.63%) | .020 | | NA | | 71 | |  |
| Ear/Nose/Throat  /Mouth | 8(3.64%) | 4(1.59%) | 3(.723%) | 6(1.17%) | .020 | | NA | | 21 | |  |
| Ophthalmology | 6(2.73%) | 17(6.75%) | 20(4.82%) | 3(.583%) | <.001 | | .182 | | 46 | |  |
| First Aid | 2(.909%) | 1(.397%) | 3(.723%) | 4(.777%) | .601 | | NA | | 10 | |  |
| Gastrointestinal | 4(1.82%) | 2(.794%) | 8(1.93%) | 9(1.75%) | .334 | | NA | | 23 | |  |
| Healthy Living | 55(25.0%) | 95(37.7%) | 156(37.6%) | 197(38.3%) | <.001 | | .128 | | 503 | |  |
| Hormone | 0(0%) | 1(.397%) | 0(0%) | 1(.194%) | .378 | | NA | | 2 | |  |
| LGBTIQ+ | 3(1.36%) | 1(.397%) | 0(0%) | 3(.583%) | .041 | | NA | | 7 | |  |
| Medicines and Clinical Reference | 23(10.5%) | 13(5.16%) | 41(9.88%) | 48(9.32%) | .037 | | NA | | 125 | |  |
| Men's Health | 0(0%) | 1(.397%) | 0(0%) | 0(0%) | .329 | | NA | | 1 | |  |
| Mental Health | 45(20.5%) | 90(35.7%) | 122(29.4%) | 144(28.0%) | <.001 | | .169 | | 401 | |  |
| Musculoskeletal | 12(5.45%) | 2(.794%) | 11(2.65%) | 20(3.88%) | .005 | | NA | | 45 | |  |
| Neurodiverse | 8(3.64%) | 8(3.17%) | 7(1.69%) | 20(3.88%) | .051 | | NA | | 43 | |  |
| Neurological | 19(8.64%) | 15(5.95%) | 26(6.27%) | 47(9.13%) | .158 | | NA | | 107 | |  |
| Pain Management | 6(2.73%) | 4(1.59%) | 10(2.41%) | 14(2.72%) | .526 | | NA | | 34 | |  |
| Pregnancy | 7(3.18%) | 10(3.97%) | 25(6.02%) | 37(7.18%) | .041 | | NA | | 79 | |  |
| Respiratory | 15(6.82%) | 0(0%) | 9(2.17%) | 37(7.18%) | <.001 | | .158 | | 61 | |  |
| Sexual Health | 3(1.36%) | 14(5.56%) | 22(5.30%) | 14(2.72%) | .023 | | NA | | 53 | |  |
| Dermatology | 7(3.18%) | 6(2.38%) | 7(1.69%) | 7(1.36%) | .137 | | NA | | 27 | |  |
| Social Prescribing | 2(.909%) | 0(0%) | 1(.241%) | 3(.583%) | .217 | | NA | | 6 | |  |
| Social support network | 4(1.82%) | 3(1.19%) | 3(.723%) | 5(.971%) | .243 | | NA | | 15 | |  |
| Tropical diseases | 0(0%) | 0(0%) | 0(0%) | 2(.388%) | .505 | | NA | | 2 | |  |
| Urology | 4(1.82%) | 0(0%) | 1(.241%) | 8(1.55%) | .047 | | NA | | 13 | |  |
| Utilities/ Administration | 8(3.64%) | 7(2.78%) | 9(2.17%) | 18(3.50%) | .306 | | NA | | 42 | |  |
| Women's Health | 6(2.73%) | 14(5.56%) | 21(5.06%) | 18(3.50%) | .170 | | NA | | 59 | |  |
| Workforce monitoring | 0(0%) | 0(0%) | 1(.241%) | 4(.777%) | .309 | | NA | | 5 | |  |

**Appendix 4 Table 2**: Category per cluster, category assignment is mutually inclusive. One app has not been assigned to any category. Percentages (%) are of category per cluster sample size. Bonferroni corrected alpha .05/33 ≈.002.

*Fisher exact test *p*-value between largest (red) and smallest (yellow) relative cluster percentage.

**Appendix 4 Table 3**: Features per cluster, feature assignment is mutually inclusive. Percentages (%) are of feature per cluster sample size. Bonferroni corrected alpha .05/19 ≈ .003.

|  | **Cluster** | | | |  |  |  |
| --- | --- | --- | --- | --- | --- | --- | --- |
| **Feature** | Apps with poor user rating  (n=220) | Apps with poor PCA/DP  (n=252) | Apps with poor PCA  (n=415) | Higher quality apps with higher user ratings  (n=515) | ***p*-value** | **Cohen W** | **Total** |
| Behavioural Change Techniques | 11(5.00%) | 12(4.76%) | 26(6.27%) | 32(6.21%) | .493 | NA | 81 |
| Condition Management | 92(41.8%) | 84(33.3%) | 138(33.3%) | 154(29.9%) | .002 | .116 | 468 |
| Data Capture | 215(97.7%) | 252(100%) | 399(96.1%) | 504(97.9%) | <.001 | .122 | 1370 |
| Data Sharing | 213(96.8%) | 252(100%) | 385(92.8%) | 495(96.1%) | <.001 | .169 | 1345 |
| Diagnostic Support | 6(2.73%) | 5(1.98%) | 13(3.13%) | 7(1.36%) | .072 | NA | 31 |
| Environmental Data | 6(2.73%) | 7(2.78) | 20(4.82%) | 15(2.91%) | .292 | NA | 48 |
| Goal Setting and Gamification | 72(32.7%) | 84(33.3%) | 158(38.1%) | 247(48.0%) | <.001 | .141 | 561 |
| Health Monitoring | 71(32.3%) | 94(37.3%) | 175(42.2%) | 210(40.8%) | .017 | NA | 550 |
| Information Provision | 212(96.4%) | 251(99.6%) | 412(99.3%) | 510(99.0%) | .014 | NA | 1385 |
| Online Consultation | 22(10.0%) | 17(6.75%) | 29(6.99%) | 69(13.4%) | .007 | NA | 137 |
| Online Prescriptions | 2(.909%) | 0(0%) | 1(.241) | 8(1.55%) | .058 | NA | 11 |
| Personal Health Record | 4(1.82%) | 0(0%) | 0(0%) | 5(.971%) | .014 | NA | 9 |
| Remote Clinical Monitoring | 24(10.9%) | 17(6.75%) | 28(6.75%) | 31(6.02%) | .031 | NA | 100 |
| Remote Monitoring | 53(24.1%) | 27(10.7%) | 62(14.9%) | 110(21.4%) | <.001 | .178 | 252 |
| Risk Indicator | 21(9.55%) | 29(11.5%) | 47(11.3%) | 43(8.35%) | .187 | NA | 140 |
| Service Signposting | 72(32.7%) | 32(12.7%) | 78(18.8%) | 140(27.2%) | <.001 | .241 | 322 |
| Treatment Delivery | 1(.455%) | 0(0%) | 8(1.93%) | 3(.583%) | .028 | NA | 12 |
| Treatment Support | 5(2.27%) | 5(1.98%) | 13(3.13%) | 13(2.52%) | .465 | NA | 36 |
| Utility / Administrative | 4(1.82%) | 0(0%) | 0(0%) | 7(1.36%) | .014 | NA | 11 |

*Fisher exact test *p*-value between largest (red) and smallest (yellow) relative cluster percentage.
